# Supplementary material for: Inducing and tuning Kondo screening in a narrow-electronic-band system
Source: Nat Commun. 2022 Apr 20;13:2156. doi: 10.1038/s41467-022-29891-4 (PMC9021272; doi:10.1038/s41467-022-29891-4)
Supplement: Supplementary file 1 — Supplementary Information [file 41467_2022_29891_MOESM1_ESM.pdf]

# SUPPLEMENTARY INFORMATION

# Inducing and tuning Kondo screening in a narrow-electronic-band system

Shiwei Shen[1], Chenhaoping Wen[1], Pengfei Kong[1], Jingjing Gao[2,3], Jianguo Si[2], Xuan Luo[2], Wenjian Lu[2], Yuping Sun[2,4,5], Gang Chen[6], Shichao Yan[1,7,*]

[1]*School of Physical Science and Technology, ShanghaiTech University, Shanghai 201210, China*

[2]*Key Laboratory of Materials Physics, Institute of Solid State Physics, HFIPS, Chinese Academy of Sciences, Hefei 230031, China*

[3]*University of Science and Technology of China, Hefei 230026, China*

[4]*High Magnetic Field Laboratory, HFIPS, Chinese Academy of Sciences, Hefei 230031, China*

[5]*Collaborative Innovation Centre of Advanced Microstructures, Nanjing University, Nanjing 210093, China*

[6]*Department of Physics and HKU-UCAS Joint Institute for Theoretical and Computational Physics at Hong Kong, The University of Hong Kong, Hong Kong, China*

[7]*ShanghaiTech Laboratory for Topological Physics, ShanghaiTech University, Shanghai 201210, China*

**Email: yanshch@shanghaitech.edu.cn*

## Contents

1. **Supplementary Note 1: Large-area STM topographies on the Type-I and Type-II 1*T*-$TaS_2$ surfaces with Pb islands**
2. **Supplementary Note 2: Structures of the Pb islands on the Type-I and Type-II surfaces**
3. **Supplementary Note 3: STM topographies taken on the Type-II surface with positive and negative bias voltages**
4. **Supplementary Note 4: Changing the position of the SD cluster during the STM measurements**
5. **Supplementary Note 5: The boundary between the Type-I and the Type-II surface terminations**
6. **Supplementary Note 6: Comparison with the d*I*/d*V* spectra taken on the pristine Type-I and Type-II 1*T*-$TaS_2$ surfaces**
7. **Supplementary Note 7: Fourier transform images of the STM topographies taken on the Type-II surface with intercalated Pb atoms**
8. **Supplementary Note 8: d*I*/d*V* spectra taken on the Pb adatoms adsorbed on the Type-II surface**
9. **Supplementary Note 9: Spatial variation of the Kondo resonance peak shown in Fig. 2e**
10. **Supplementary Note 10: Comparison with the Kondo resonance peak in the single layer 1*T*-$TaS_2$ grown on single layer 1*H*-$TaS_2$**
11. **Supplementary Note 11: First-principles calculations for intercalated Pb atoms below the Type-II surface**
12. **Supplementary Note 12: STM topographies taken on the regions with different intercalant Pb concertation**
13. **Supplementary Note 13: Evolution of the d*I*/d*V* spectra in the dark SD clusters as increasing the concentration of the dark SD clusters.**
14. **Supplementary Note 14: More d*I*/d*V* maps taken on the same region as shown in Fig. 5a**
15. **Supplementary References**

**Supplementary Note 1: Large-area STM topographies on the Type-I and Type-II 1*T*-$TaS_2$ surfaces with Pb islands**

For the cleaved 1*T*-$TaS_2$ sample, the Type-I surface is the most common surface termination. After evaporating Pb atoms, both the Type-I and Type-II surface terminations can be observed in the room-temperature cleaved 1*T*-$TaS_2$ sample. The Pb atoms may disturb the stacking between the topmost 1*T*-$TaS_2$ and the underneath 1*T*-$TaS_2$, which would be helpful for the formation of the Type-II surface. Pb atoms form regular islands on both the Type-I and Type-II surfaces. In the large-area STM topographies taken on both Type-I and Type-II surfaces, we find a few CDW domain walls (as shown by the yellow dashed lines in Figs. S1a and S1b). These CDW domain walls often connect the Pb islands and are likely pinned by these Pb islands. This indicates the Pb islands can induce a few CDW domain walls, but the CDW domain walls are not directly related with the intercalated Pb atoms. On both Type-I and Type-II surfaces with Pb islands, we can find single-CDW-domain regions with the sizes of a few tens of nanometers.

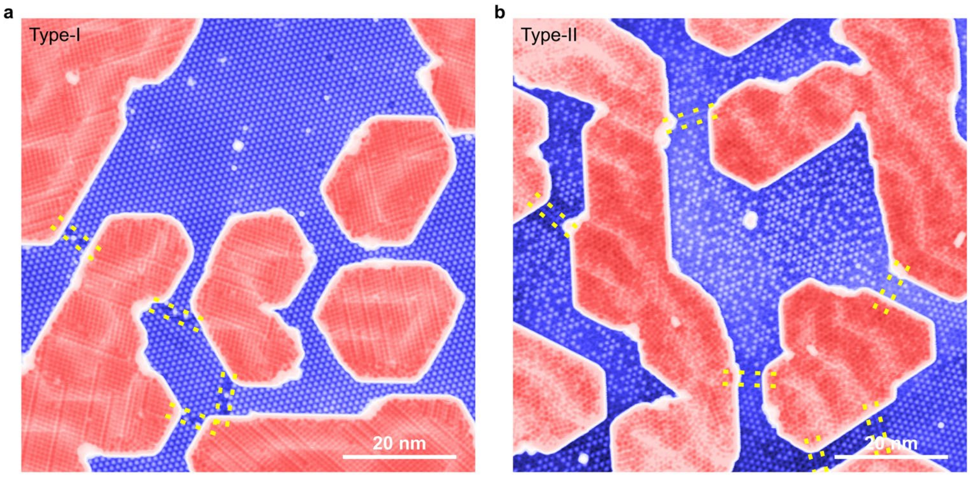


**Figure S1 a,** Constant-current STM topography taken on the Type-I 1*T*-$TaS_2$ surface with Pb islands. **b,** Constant-current STM topography taken on the Type-II 1*T*-$TaS_2$ surface with Pb islands. The yellow dashed lines indicate the CDW domain walls.

**Supplementary Note 2: Structures of the Pb islands on the Type-I and Type-II surfaces**

As shown in the Fig. S2, the Pb islands appear different on the Type-I surface and the Pb intercalated Type-II surface. However, as we can also see from Fig. 2a in the main text, for the Type-II surface, when the Pb islands are grown on the small region where there are no dark SD clusters and with ~50 mV insulating gap, the Pb islands appear similar as that on the Type-I surface. It is very likely that the Pb islands have different structures when they are grown on the insulating and the metallic 1*T*-$TaS_2$ surfaces. The periodic height modulation in the Pb islands may be due to the strain effect.

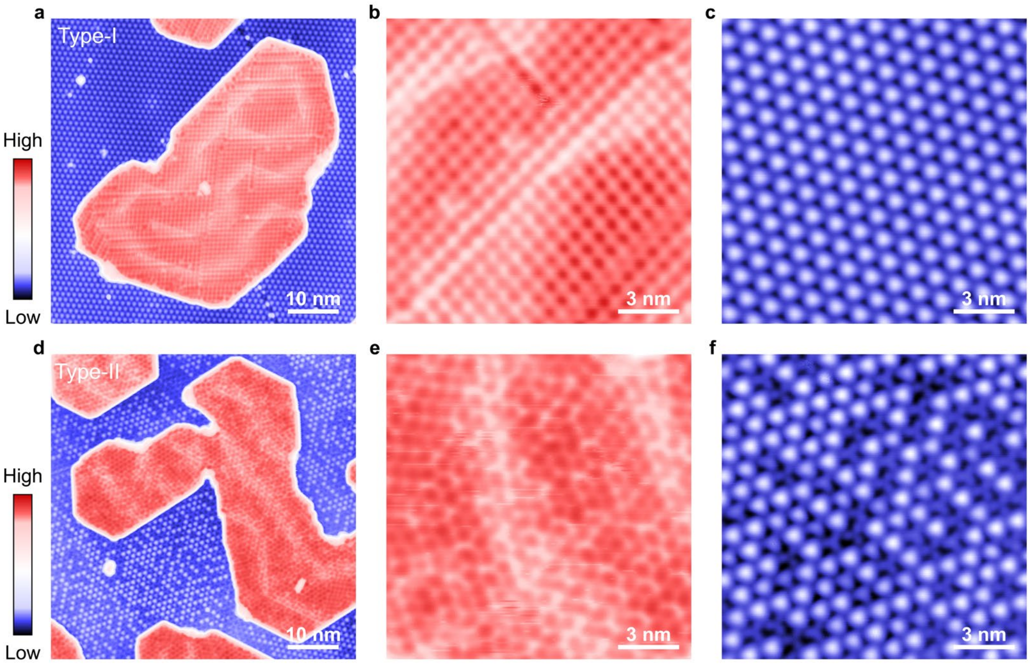


**Figure S2 a,** Constant-current STM topography taken on the Type-I 1*T*-$TaS_2$ surface with Pb islands ($V_s$ = −500 mV, $I$ = 20 pA). **b, c,** Zoom-in views of the Pb island and the bare Type-I surface, respectively ($V_s$ = −500 mV, $I$ = 100 pA). **d,** Constant-current STM topography taken on Type-II 1*T*-$TaS_2$ surface with Pb islands ($V_s$ = −500 mV, $I$ = 20 pA). **e, f,** Zoom-in views of the Pb island and the bare Type-II surface, respectively ($V_s$ = −500 mV, $I$ = 20 pA).

**Supplementary Note 3: STM topographies taken on the Type-II surface with positive and negative bias voltages**

As we can see from the below STM topographies taken on the same region of the Type-II surface with negative and positive bias voltages, the dark SD clusters are less visible in the positive-bias-voltage STM topography. As shown by the dashed black lines in Fig. S3a, we can still find a small region on the Type-II surface where there are no dark SD clusters.

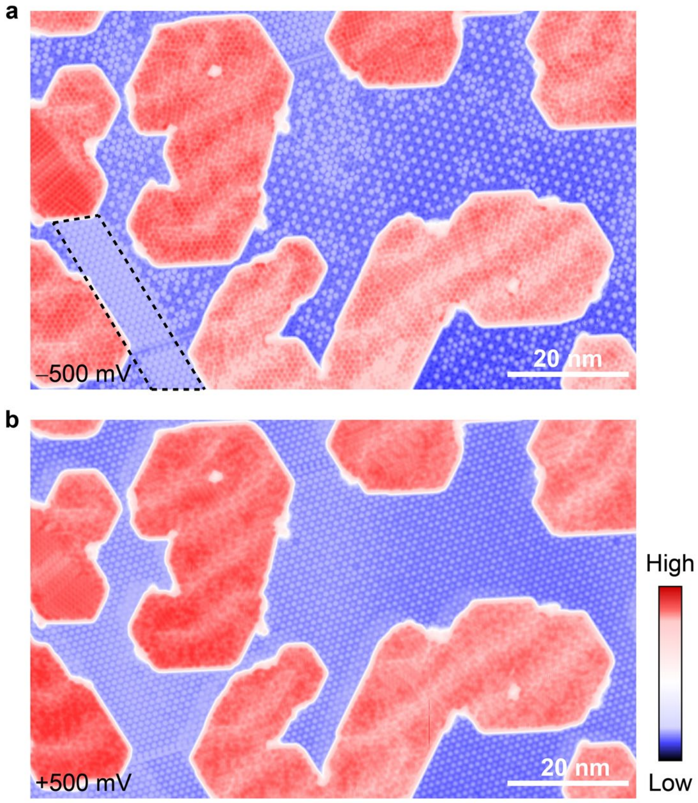


**Figure S3 a,** Constant-current STM topography taken on the Type-II 1*T*-$TaS_2$ surface with Pb islands taken with negative bias voltage ($V$s = −500 mV, $I$ = 20 pA). **b,** Constant-current STM topography taken on the same region as (a) with positive bias voltage ($V$s = 500 mV, $I$ = 20 pA).

**Supplementary Note 4: Changing the position of the SD cluster during the STM measurements**

Although the surface adatoms can be manipulated by STM tip, manipulating the intercalated atoms below the topmost layer in STM experiments is difficult. We cannot controllably manipulate the intercalated Pb atoms, but the position of the dark cluster can indeed be changed but rarely during the STM measurements. As shown in the STM topographies in Fig. S4, after performing a few d$I$/d$V$ measurements in this region, the dark SD cluster marked by the purple circle is changed while the dark SD cluster marked by the green circle remains unchanged. This indicates that the position of the dark SD cluster can be changed by the STM tip.

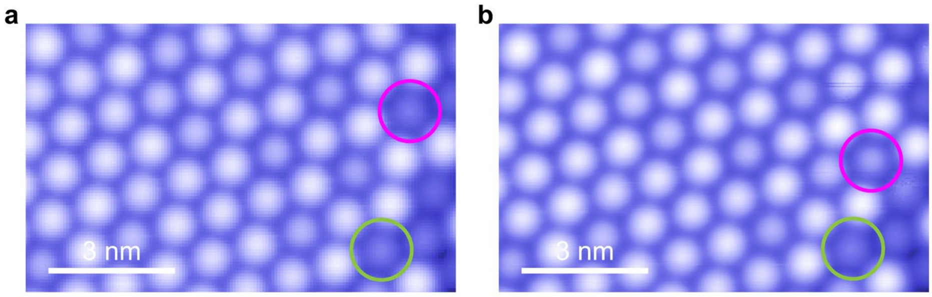


**Figure S4** STM topographies taken before (a) and after (b) performing the d$I$/d$V$ measurements in this region (a: $V_s = -200$ mV, $I = 20$ pA; b: $V_s = -200$ mV, $I = 20$ pA). The purple and green circles indicate the positions of two dark SD clusters.

**Supplementary Note 5: The boundary between the Type-I and the Type-II surface terminations**

Both the Type-I and the Type-II surfaces can be seen on the same sample. As shown in Fig. S5, all the SD clusters in the Type-I surface appear as bright SD clusters and the ~150 mV insulating gap still can be seen on the bare Type-I surface. This indicate that the dark SD clusters on the bare Type-II surface are not induced by the intrinsic impurities in the 1*T*-$TaS_2$ sample. The positions of the dark SD clusters can be changed during STM measurements (Fig. S4). On the other hand, because the height of the Pb adatoms is about ~230 pm (Fig. S8), the dark SD clusters are also not induced by the Pb adatoms on the surface. The only possibility for inducing the dark SD clusters is there are intercalated Pb atoms underneath the dark SD clusters.

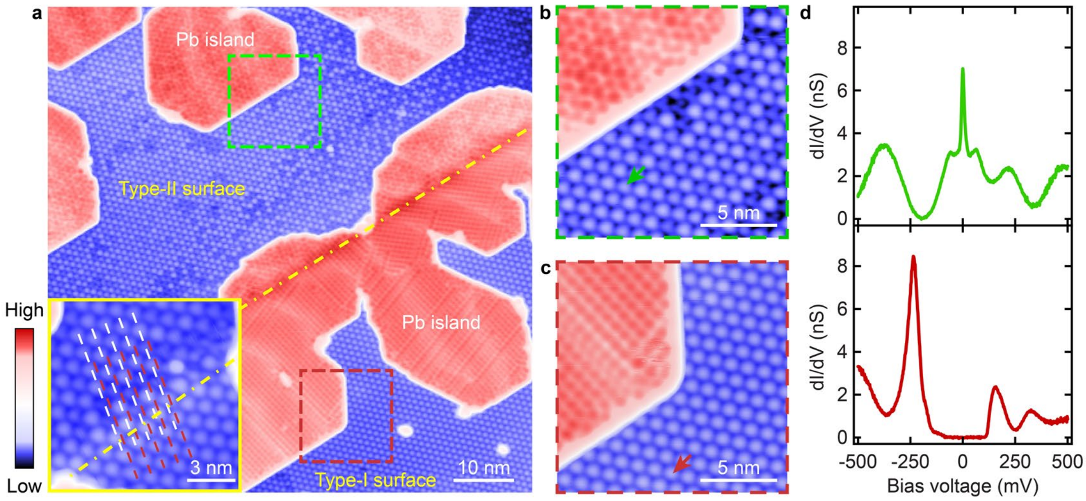


**Figure S5 a,** Constant-current STM topography taken near the boundary between the Type-I and Type-II 1*T*-$TaS_2$ surfaces with Pb islands ($V$s = −500 mV, $I$ = 20 pA). The insert is the zoom-in view near the boundary between the Type-I and Type-II surfaces. **b, c,** Constant-current STM topographies taken on green and red dashed squares, respectively ($V$s = −500 mV, $I$ = 20 pA). **d,** d$I$/d$V$ spectra taken on spots marked with the green and red arrows in (b) and (c).

**Supplementary Note 6: Comparison with the d*I*/d*V* spectra taken on the pristine Type-I and Type-II 1*T*-$TaS_2$ surfaces**

As we can see from Fig. S6a, the size of the insulating gap (~150 mV) on the bare Type-I surface is the same as that on the pristine Type-I surface. As shown in Fig. S6b, the size of the insulating gap on the bare Type-II 1*T*-$TaS_2$ surface (~50 mV) where there are no dark SD clusters is similar as that on the pristine Type-II surface. The d*I*/d*V* spectra on the pristine Type-I and Type-II surfaces are from *Nat. Commun.* **11**, 2477 (2020).

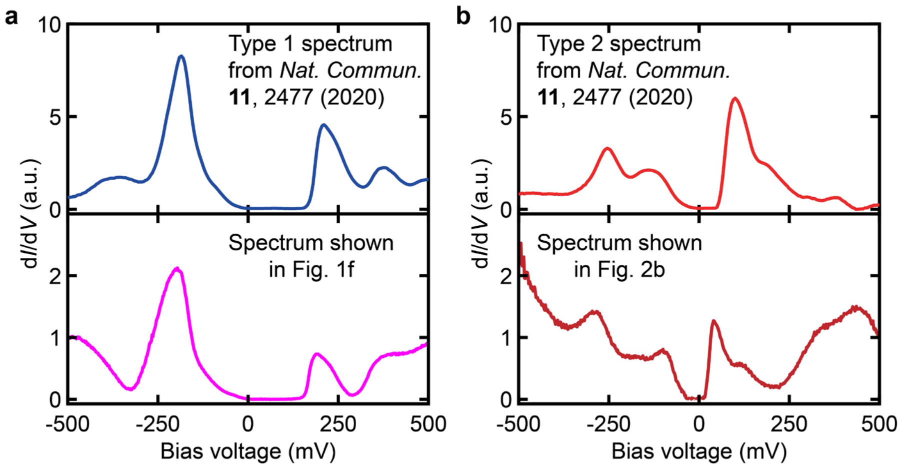


**Figure S6 a,** Top panel: d*I*/d*V* spectrum taken on the pristine Type-I 1*T*-$TaS_2$ surface (from *Nat. Commun.* **11**, 2477 (2020)). Bottom panel: d*I*/d*V* spectrum taken on the bare Type-I 1*T*-$TaS_2$ surface. **b,** Top panel: d*I*/d*V* spectrum taken on the pristine Type-II 1*T*-$TaS_2$ surface (from *Nat. Commun.* **11**, 2477 (2020)). Bottom panel: d*I*/d*V* spectrum taken on the small region of the bare Type-II surface where there are no dark SD clusters.

**Supplementary Note 7: Fourier transform images of the STM topographies taken on the Type-II surface with intercalated Pb atoms**

In order to demonstrate the influence of the intercalated Pb atoms to the long-range CDW order in 1*T*-$TaS_2$, we perform Fourier transform (FT) to the STM topographies shown in Figs. 1g and 2a (Figs. S7b and S7e). The yellow circles in Figs. S7b and S7e indicate the CDW wavevectors. We then perform inverse Fourier transform (IFT) by filtering the CDW wavevectors (Figs. S7c and S7f). As shown in Figs. S7c and S7f, the CDW order in the bare 1*T*-$TaS_2$ region is long-range ordered without CDW domain walls.

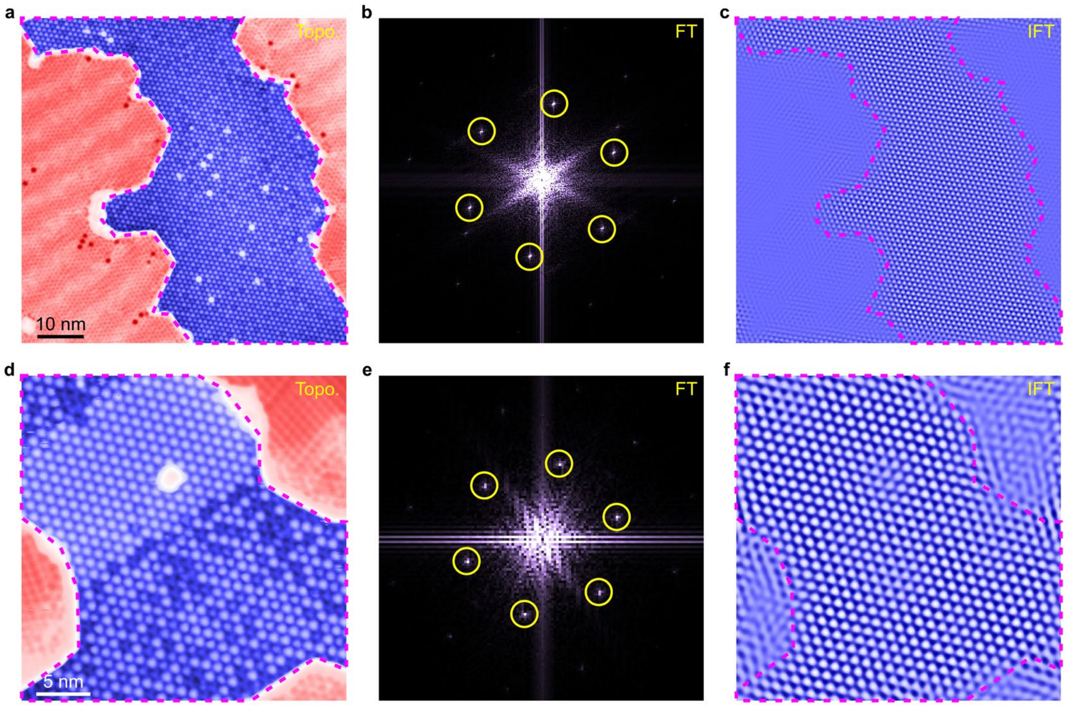


**Figure S7 a**, STM topography shown in Fig. 1g. **b**, FT of a. **c**, The IFT by filtering the CDW wavevectors in the yellow circles in **b**. **d-f**, The same as a-c, but performed for STM topography shown in Fig. 2a. The yellow circles indicate the CDW wavevectors in **b** and **e**. The purple dashed lines in **a**, **c**, **d**, and **e** indicate the single long-range CDW domain region.

## Supplementary Note 8: d*I*/d*V* spectra taken on the Pb adatoms adsorbed on the Type-II surface

On the Type-II surface, except the dark SD clusters, we can also see a few Pb adatoms. The height of the Pb adatoms is about ~230 pm. Although there is strong Kondo resonance on the bare Type-II surface with intercalated Pb atoms, no Kondo peak is detected in the d*I*/d*V* spectra taken on the Pb adatoms.

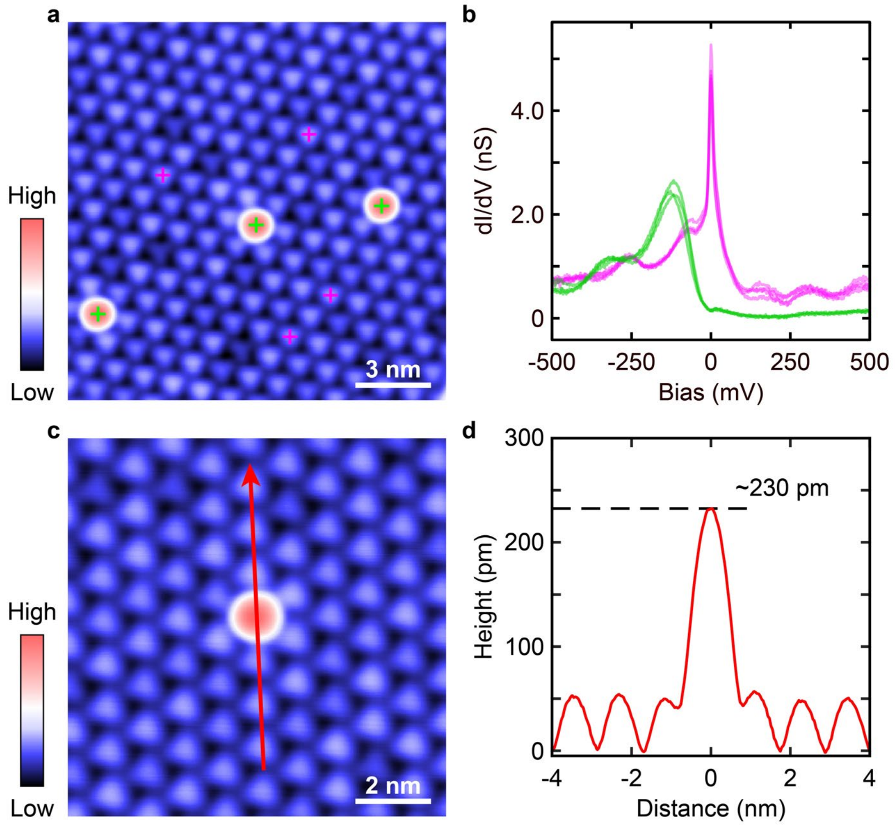


**Figure S8 a,** Constant-current STM topography on the Type-II 1*T*-$TaS_2$ surfaces with a few Pb adatoms with ~230 pm height ($V$s = −500 mV, $I$ = 30 pA). **b,** d*I*/d*V* spectra taken on the spots marked with the green and purple crosses in (a). **c,** Constant-current STM topography on Type-II 1*T*-$TaS_2$ surfaces with a Pb adatom ($V$s = −500 mV, $I$ = 30 pA). **d,** Line profile along the red arrow shown in (c).

**Supplementary Note 9: Spatial variation of the Kondo resonance peak shown in Fig. 2e**

Fig. 2e shows the d*I*/d*V* spectra taken along the red arrow shown in Fig. 2c. In order to show the spatial variation of the ZBP shown in Fig. 2c, we have fitted and plotted the width of the ZBP in each d*I*/d*V* spectrum shown in Fig. 2c. As we can see from Fig. S9, the width of the ZBPs in the bright SD clusters has very little spatial variation.

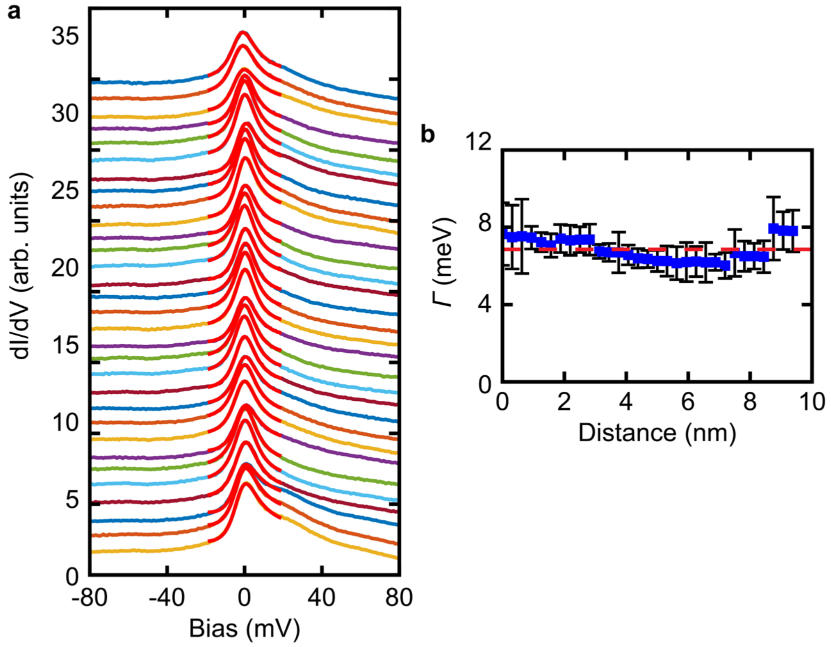


**Figure S9** **a**, d*I*/d*V* spectra shown in Fig. 2e. The spectra are vertically offset for clarity. The red lines are the thermally convolved Fano fits to the ZBP. **b**, The half width ($\Gamma$) at half maximum of the d*I*/d*V* spectra shown in **a**. The red dashed line is the averaged $\Gamma$.

**Supplementary Note 10: Comparison with the Kondo resonance peak in the single layer 1*T*-$TaS_2$ grown on single layer 1*H*-$TaS_2$**

Although no clear splitting is observed in the Kondo resonance peak with external magnetic field up to 8.5 T (Fig. 3c), the Kondo resonance peak in single layer 1*T*-$TaS_2$ is split with 10 T magnetic field (Ref. 20). The difference is that the width of Kondo resonance peak in Fig. 3c is about four times the width of the Kondo resonance peak in single layer 1*T*-$TaS_2$ (as shown in Fig. S10). This makes the Kondo resonance peak in Fig. 3c less sensitive to the splitting induced by the magnetic field, and larger magnetic field is needed to induce the splitting of the Kondo resonance peak in Pb intercalated Type-II surface.

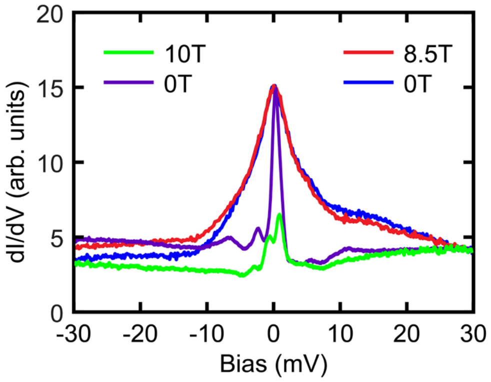


**Figure S10** The d*I*/d*V* spectra taken with 0 T (blue) and 8.5 T (red) magnetic fields shown in Fig. 3c. The d*I*/d*V* spectra taken on the 1*T*-$TaS_2$ layer in the 1*T*/1*H*-$TaS_2$ heterostructure with 0 T (purple) and 10 T (green) magnetic fields (from Ref. 20).

**Supplementary Note 11: First-principles calculations for intercalated Pb atoms below the Type-II surface**

The first-principles calculations were performed based on density functional theory (DFT) by using the Vienna ab initio simulation package (VASP)[1,2]. The generalized gradient approximation (GGA) with the Perdew-Burke-Ernzerhof (PBE) parametrization[3,4] was used to describe the exchange-correlation interaction. In order to treat the van der Waals interaction, the Tkatscenko-Scheffler scheme[5] was used. The plane-wave cutoff energy was set to be 300 eV and a 4×4×4 *k*-point mesh was used. We construct the superstructures with Type-II stacking and intercalate the Pb atom below the Type-II 1*T*-$TaS_2$ layer. The crystal structures were fully relaxed until all the residual forces were less than $10^{-3}$ eV/Å. Charge transfer was evaluated by Bader charge analysis[6]. The charge analysis shows that ~1.1 electrons transfer from the Pb atom to the adjacent 1*T*-$TaS_2$ layers. For Type-II stacking, the transferred electrons are symmetrically distributed in two adjacent 1*T*-$TaS_2$ layers.

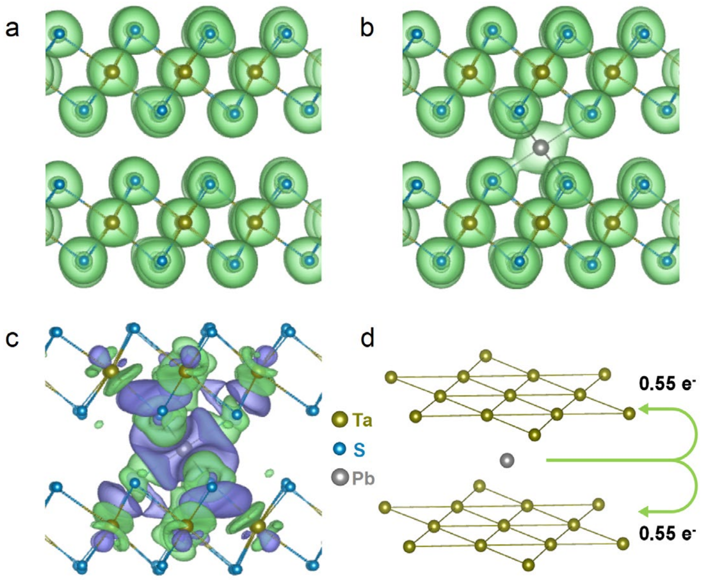


**Figure S11** Side view of charge densities for 1*T*-$TaS_2$ (**a**) and Pb intercalated below the Type-II 1*T*-$TaS_2$ surface (**b**). Isosurface value is 0.1 e/bohr$^3$. **c**, Side view of differential charge densities for the intercalated Pb atom below the Type-II 1*T*-$TaS_2$ surface. Isosurface value is 0.002 e/bohr$^3$. Green and purple contours indicate charge accumulation and reduction, respectively. **d**, Schematic diagram of the charge transfer of Pb atom to the adjacent 1*T*-$TaS_2$ layers. Here for convenient view only Ta atoms are shown in the corresponding 1*T*-$TaS_2$ layers.

**Supplementary Note 12: STM topographies taken on the regions with different intercalant Pb concertation**

As shown in the below STM topographies, we can see the individual randomly distributed dark SD clusters with 5 minutes room-temperature annealing (Fig. S12a). When we perform room-temperature annealing for 10 minutes, the density of the dark SD clusters becomes higher (Fig. S12b). However, after 10 minutes room-temperature annealing, the spatial distribution of the dark SD clusters is not very uniform. As shown in Figs. S12c-S12f, we can find different intercalant concentration of Pb in different areas.

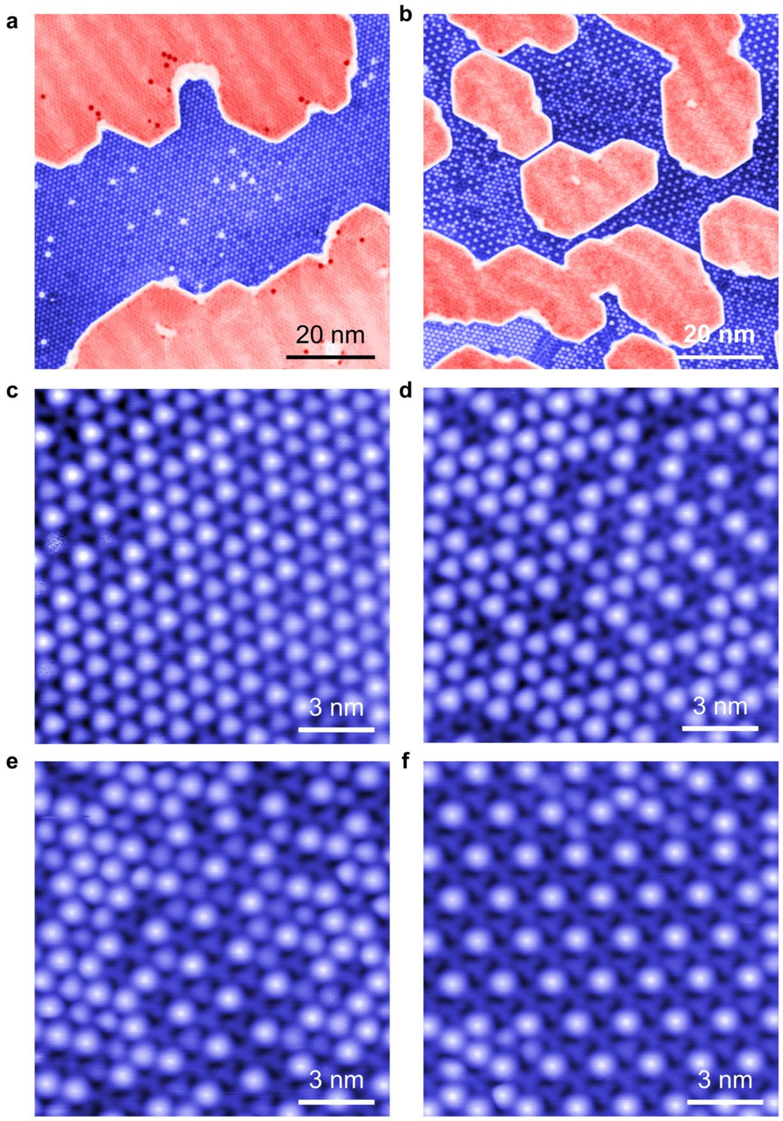


**Figure S12 a**, STM topography taken with 5 minutes room-temperature annealing after evaporating Pb atoms. **b**, STM topography taken with 10 minutes room-temperature annealing after evaporating Pb atoms. **c-f,** Constant-current STM topographies taken on the Pb intercalated Type-II 1*T*-$TaS_2$ surface with different Pb concentration ($V$s = −500 mV, $I$ = 20 pA).

**Supplementary Note 13: Evolution of the d*I*/d*V* spectra in the dark SD clusters as increasing the concentration of the dark SD clusters.**

As shown in Fig. S13, when the intercalant concentration of the Pb atoms increases, the narrow electronic band is shifted to be above the Fermi level.

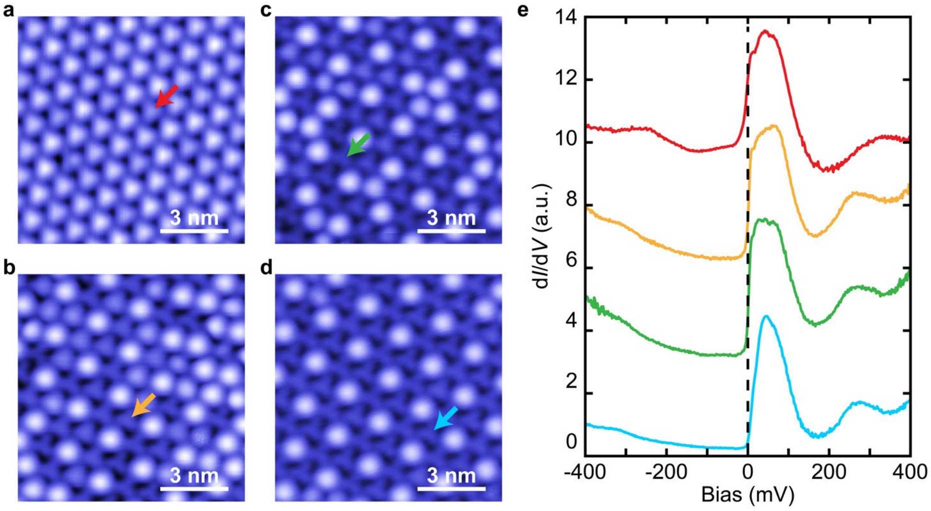


**Figure S13 a-d**, Constant-current STM topographies taken on the Type-II 1*T*-$TaS_2$ surface as increasing the intercalant concentration of Pb. **e**, d*I*/d*V* spectra taken on the colored arrows shown in (**a-d**).

**Supplementary Note 14: More d*I*/d*V* maps taken on the same region as shown in Fig. 5a**

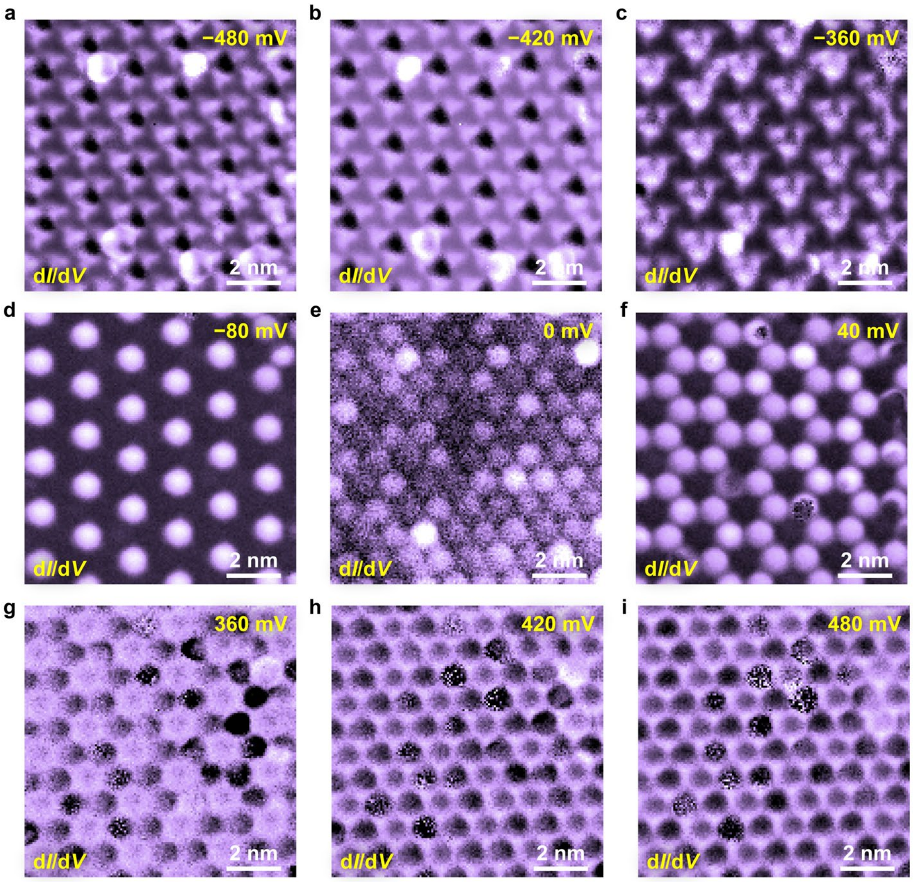


**Figure S14 a-i**, d*I*/dV maps taken on the same region as shown in Fig. 5a with −480 mV (a), −420 mV (b), −360 mV (c), −80 mV (d), 0 mV (e), 40 mV (f), 360 mV (g), 420 mV (h), and 480 mV (i) bias voltages, respectively.

**Supplementary References**


1. Kresse G., Hafner J., *Phys. Rev. B* **48**, 13115 (1993).
2. Kresse G., Furthmüller J., *Compos. Mater. Sci.* **6**, 15 (1996).
3. Blöchl P. E., *Phys. Rev. B* **50**, 17953 (1994).
4. Perdew J. P., Burke K., Ernzerhof M., *Phys. Rev. Lett.* **77**, 3865 (1996).
5. Tkatchenko A., Scheffler M., *Phys. Rev. Lett.* **102**, 073005 (2009).
6. Tang W., Sanville E., Henkelman G., *J. Phys.: Condens. Matter* **21**, 084204 (2009).
7. Momma K., Izumi F., *J. Appl. Cryst.* **44**, 1272 (2011).
